# A bell‐shaped pattern of urinary aquaporin‐2‐bearing extracellular vesicle release in an experimental model of nephronophthisis

**DOI:** 10.14814/phy2.14092

**Published:** 2019-05-09

**Authors:** Nobuyuki Mikoda, Hiroko Sonoda, Sayaka Oshikawa, Yuya Hoshino, Toshiyuki Matsuzaki, Masahiro Ikeda

**Affiliations:** ^1^ Department of Veterinary Pharmacology University of Miyazaki Miyazaki Japan; ^2^ Department of Anatomy and Cell Biology Gunma University Graduate School of Medicine Maebashi Japan

**Keywords:** Aquaporin‐2, pcy mice, polycystic kidney disease, urinary extracellular vesicles (exosomes)

## Abstract

The DBA/2‐FG pcy (pcy) mouse is a model of human nephronophthisis, a recessive cystic kidney disease. Renal expression of aquaporin‐2 (AQP2), a water channel protein, has been shown to be altered in pcy mice. However, the relationship between the renal expression and its release in urinary extracellular vesicles (uEV‐AQP2), which account for most urinary AQP2, in pcy mice has remained largely unknown. In this study, we examined age‐related alterations of this relationship in pcy mice. In comparison with control mice, pcy mice after the age of 14 weeks showed defective urinary concentration ability with an increase in urinary volume. Interestingly, the release of uEV‐AQP2 increased progressively up to the age of 16 weeks, but at 21 weeks the release did not significantly differ from that in control mice (i.e., a bell‐shaped pattern was evident). Similar results were obtained for uEV marker proteins, including tumor susceptibility gene 101 (TSG101) protein and apoptosis‐linked gene 2‐interacting protein X (Alix). Immunoblot analysis revealed that renal AQP2 expression increased progressively from 11 weeks, and immunohistochemistry showed that this increase was possibly due to an increase in the number of AQP2‐positive cells. Analysis of mRNAs for seven types of AQP expressed in the kidney supported this notion. These data suggest that the level of uEV‐AQP2 does not simply mirror the renal expression of AQP2 and that the altered release of uEV‐AQP2 in pcy mice depends on the numbers of both renal AQP2‐positive cells and EVs released into the urine.

## Introduction

Nephronophthisis (NPHP), characterized by formation of multiple cysts in the kidney, constitutes the commonest genetic cause of end‐stage renal failure in the first three decades of life (Krishnan et al. 2008; Hildebrandt et al. 2009; Luo and Tao 2018). So far, more than 20 genes responsible for NPHP have been identified, including NPHP1, INVS, NPHP3, NPHP4, IQCB1, CEP290, and GLIS2 (Luo and Tao 2018). DBA/2‐FG pcy (pcy) mice have been shown to harbor a mutation in the Nphp3 gene (Olbrich et al. 2003), and therefore, these mice have been considered an experimental model of NPHP (Hildebrandt et al. 2009; Luo and Tao 2018).

Urinary extracellular vesicles (uEVs), including exosomes and microvesicles, have attracted attention over the last decade because of recognition of uEVs as communication mediators and a source of novel biomarkers (Pisitkun et al. 2004; Huebner et al. 2015; Oshikawa et al. 2016; Zhang et al. 2016). Exosomes, 30–150 nm in diameter, are generated through fusion of multivesicular bodies with the apical membrane of renal epithelial cells. Microvesicles, 50–2000 nm in diameter, are derived from a process of budding from the renal apical membrane. These uEVs have been shown to include many candidate biomarkers for kidney disease, such as miR‐26a (lupus nephritis), miR‐29, CD2AP mRNA, IL‐18 mRNA (chronic kidney disease), aquaporin‐1 protein (AQP1), AQP2, NKCC2 protein, NCC protein (tubule damage or tubulopathy), and so on (Huebner et al. 2015; Oshikawa et al. 2016; Pomatto et al. 2017; Sonoda et al. 2019).

So far, material isolated using any of the currently available methods has been shown to contain a mixture of exosomes and microvesicles (Colombo et al. 2014). Therefore, in this paper, we use the term EVs instead of exosomes and microvesicles.

AQPs are important proteins for water handling in the kidney, and at least seven isoforms (AQP1, AQP2, AQP3, AQP4, AQP6, AQP7, and AQP11) are known to be expressed in this tissue (Nielsen et al. 2002; Takata et al. 2004; Ikeda and Matsuzaki 2015). Among them, AQP2 has been found to be present in uEVs (Kanno et al. 1995; Wen et al. 1999; Abdeen et al. 2014). AQP2 is a vasopressin‐dependent water channel expressed in the principal cells of collecting ducts (Nielsen et al. 2002; Ikeda and Matsuzaki 2015). After activation of the intracellular cAMP‐protein kinase A pathway by binding of vasopressin to the V2 receptor on principal cells, AQP2 is rapidly trafficked to the apical membrane from intracellular vesicles and accumulates in the apical membrane, leading to an acute increase of water reabsorption. Also, activation of the V2 receptor enhances the level of AQP2 expression through increased transcription of the AQP2 gene, contributing to maximization of urinary concentrating capacity (Nielsen et al. 2002; Ikeda and Matsuzaki 2015).

It has been reported that pcy mice have an increased level of renal AQP2 mRNA (Gattone et al. 2003). On the other hand, AQP2 in the urine was recently reported to be predominantly localized to uEVs (Miyazawa et al. 2018). However, the feature of AQP2 release in uEVs (uEV‐AQP2) in pcy mice remains largely unknown. In the present study, in order to clarify the relationship between renal expression of AQP2 and release of uEV‐AQP2 in pcy mice, we investigated age‐related alterations in the two parameters.

## Materials and Methods

### Animals

pcy mice were bred at Kyudo Co., Ltd. (Saga, Japan). DBA/2 (DBA) mice, as age‐matched controls, were purchased from Clea Japan, Inc. (Tokyo, Japan), as DBA mice have been used as controls for pcy mice in several previous studies (Masunaga et al. 1991; Takahashi et al. 1991; Kawano et al. 2015). C57BL/6J mice were from Kyudo Co., Ltd. The animals were kept at 23 ± 3°C with 55 ± 5% humidity. They were fed a standard diet (CE‐2, Clea Japan, Inc.) and had unlimited access to tap water. All animal studies were conducted in accordance with the experimental guidelines for animal use and care at Kyudo, Co. Ltd.

### Blood and urine analyses, and isolation of urinary exosomes

Blood was collected from the tail vein at 5, 7, 11, 14, 16, and 21 weeks of age. Plasma urea nitrogen and creatinine concentrations were measured using an auto‐analyzer (Fuji Film Medical, Tokyo, Japan).

Twenty‐hour urine samples were collected at 7, 14, 16, and 21 weeks of age using metabolic cages (Toyo‐Riko Tokyo, Japan). Just after collection, the urine was centrifuged at 1000 *g* for 10 min. A small portion of the supernatant was used for measurement of urinary osmolality and creatinine concentration using osmometers (Advanced Instruments, Inc., Norwood, MA, or Arkray, Inc., Kyoto, Japan) and an auto‐analyzer, respectively.

Using the remaining urine, we isolated the uEV‐rich fraction by sequential centrifugation as previously characterized by our group in rodent urine (Sonoda et al. 2009; Asvapromtada et al. 2018). In brief, the supernatant was centrifuged at 17,000 *g* for 15 min. The resulting supernatant was retained, while the pellet was incubated at 37°C in a solution containing 250 mmol/L sucrose, 10 mmol/L triethanolamine, 8 mmol/L Hepes, and 50 mg/mL DTT, pH 7.6. Subsequently, the pellet suspension was centrifuged at 17,000 *g* for 15 min. The supernatants from two centrifugations were mixed, and the mixed solution was ultracentrifuged at 200,000 *g* for 1 h (Optima TL Ultracentrifuge; Beckman Instruments, CA). The resulting pellet (an uEV‐rich fraction) was solubilized in a solution with protease inhibitor. This suspension was then mixed with 4 × sample buffer. These samples were kept at −80°C until use. Each uEV‐sample was loaded with the same amount of urinary creatinine for analysis of protein by immunoblotting.

### Renal protein extraction

After weighing, each whole right kidney was homogenized for 5 min at 4°C using a tissue homogenizer (BioMedical Science Inc., Tokyo, Japan). The homogenate was centrifuged at 1000 *g* for 10 min at 4°C, and the pellet was homogenized again and re‐centrifuged under the same conditions. Subsequently, the supernatants from the two centrifugations were mixed and the mixed solution was centrifuged at 200,000 *g* for 1 h. The 1000 *g* supernatant and the 200,000 *g* pellet were mixed with 4 × sample buffer. The 1000 *g* supernatant was used for detection of renal *α*‐tubulin and the 200,000 *g* pellet was used for detection of renal AQP2. For immunoblotting, each renal protein sample was loaded with the same amount of total protein.

### Immunoblot analysis

Proteins in uEVs and renal samples were separated by SDS‐PAGE, and then the separated proteins were transferred to polyvinylidene difluoride membranes. After blocking with 5% skim milk in 0.05% Tween‐Tris‐buffered saline (TTBS), the membrane was incubated with 1.5% skim milk in TTBS including a primary antibody. Thereafter, the membrane was incubated with 1.5% skim milk in TTBS including a peroxidase‐conjugated secondary antibody. For the primary antibody, anti‐AQP2 (cat no. AQP‐002; Alomone Labs, Jerusalem, Israel), anti‐tumor susceptibility gene 101 (TSG101) (cat no. ab‐125011; Abcam Inc., MA), anti‐protein and apoptosis‐linked gene 2‐interacting protein X (Alix) (cat no. sc‐49268; Santa Cruz Biotechnology Inc., Dallas, TX), or anti‐*α*‐tubulin (cat no. T5168; Sigma, St. Louis, MO) antibody was used. For the secondary antibody, anti‐mouse IgG (cat no. 1858413; Thermo Fisher Scientific Inc., Rockford, IL) or anti‐rabbit IgG (cat no. 7074; Cell Signaling Technology, Danvers, MA) was used. The resulting bands were visualized using the SuperSignal West‐Femto Chemiluminescence detection system (Thermo Fisher Scientific, Waltham, MA) and were quantified using ImageQuant TL software (GE Healthcare, Uppsala, Sweden).

### Renal RNA extraction and analysis

Total RNA was extracted from each whole left kidney using an RNeasy mini kit (Qiagen, Hilden, Germany). The total RNA concentration was measured using a spectrophotometer (NanoDrop^®^ ND‐1000 Spectrophotometer, NanoDrop Technologies, DE). Purified total RNA (1 *μ*g) was reverse‐transcribed using a iScript cDNA Synthesis kit (Bio Rad Lab., Hercules, CA). Mouse AQPs and GAPDH were amplified using a FastStart Essential DNA Green Master (Roche Diagnostics, Basel, Switzerland) employing the following primers: forward (5′‐ctacactggctgcggtatca‐3′) and reverse (5′‐gcctcctctatttgggcttc‐3′) for AQP1, forward (5′‐tagccctgctctctccattg‐3′) and reverse (5′‐tgtagaggagggaaccgatg‐3′) for AQP2, forward (5′‐ccctctggacacttggacat‐3′) and reverse (5′‐gttgacggcatagccagaat‐3′) for AQP3, forward (5′‐ttgctttggactcagcattg‐3′) and reverse (5′‐gggaggtgtgaccaggtaga‐3′) for AQP4, forward (5′‐gccatgattggaacctctgt‐3′) and reverse (5′‐atcgctgggctacagtcttg‐3′) for AQP6, forward (5′‐gcccccaggtctgtgctggagaccatac‐3′) and reverse (5′‐gcctgcaaagtggttaatgg‐3′) for AQP7, forward (5′‐ccgtgctttgacgaactctt‐3′) and reverse (5′‐tatgcagccatggaaggaa‐3′) for AQP11, forward (5′‐cgggagatacatgccagtct‐3′) and reverse (5′‐cacagctgcacaaggaagaa‐3′) for V2 receptor, and forward (5′‐aacgaccccttcattgac‐3′) and reverse (5′‐tccacgacatactcagcac‐3′) for GAPDH. The expression of each gene was detected and analyzed using a Roche LightCycler^®^96 system (Roche Diagnostics).

### Histology

For immunostaining, the sections were deparaffinized and rehydrated, and the antigen was retrieved by autoclaving at 121°C for 5 min. After consuming endogenous peroxidase with 3% H_2_O_2_ solution, the slide was incubated with a primary antibody against AQP2 (cat no. AQP‐002; Alomone Labs) or AQP3 (Matsuzaki et al. 1999) at 37°C for 1 h followed by incubation with Envision System Labeled Polymer Reagent (Dako Japan) at 37°C for 45 min. For color development of the antigen, 3, 3′‐diaminobenzidine tetrahydrochloride was used. The specimen was also counterstained with hematoxylin. All specimens were scanned using a NanoZoomer 2.0 RS virtual slide scanner (C10730‐13, Hamamatsu Photonics K.K., Shizuoka, Japan), and images of each specimen were acquired using the NDP. view2 software (U12388‐01, Hamamatsu Photonics K.K.).

The area and volume of AQP (AQP2 or AQP3)‐positive cells in each field (0.42 mm^2^) in the image were measured using the WinROOF image system (ver 5.7.2, MITANI Corporation, Tokyo, Japan). In each individual, six fields for the cortex and six fields for the medulla were analyzed. The relative AQP‐positive area was calculated by determining the ratio of the AQP‐positive area to the total area. The density of AQP expression was calculated by determining the ratio of the AQP‐positive volume to the AQP‐positive area. The mean value for DBA mice was considered to be 100%.

### Statistical analysis

Box plots were generated using the BoxPlotR (http://boxplot.tyerslab.com) (Spitzer et al. 2014). Differences between DBA and pcy mice were analyzed by Mann–Whitney *U* test using a statistical analysis program provided by Osaka University (http://www.gen-info.osaka-u.ac.jp/MEPHAS/). For correlation analysis, Spearman rank correlation coefficients were calculated using the same statistical analysis program after performing a normality test using R version 3.5.0 (https://www.R-project.org/). Differences at *P* < 0.05 were considered statistically significant.

## Results

### Age‐related changes in body weight and renal function in pcy and DBA mice

**Figure 1:**
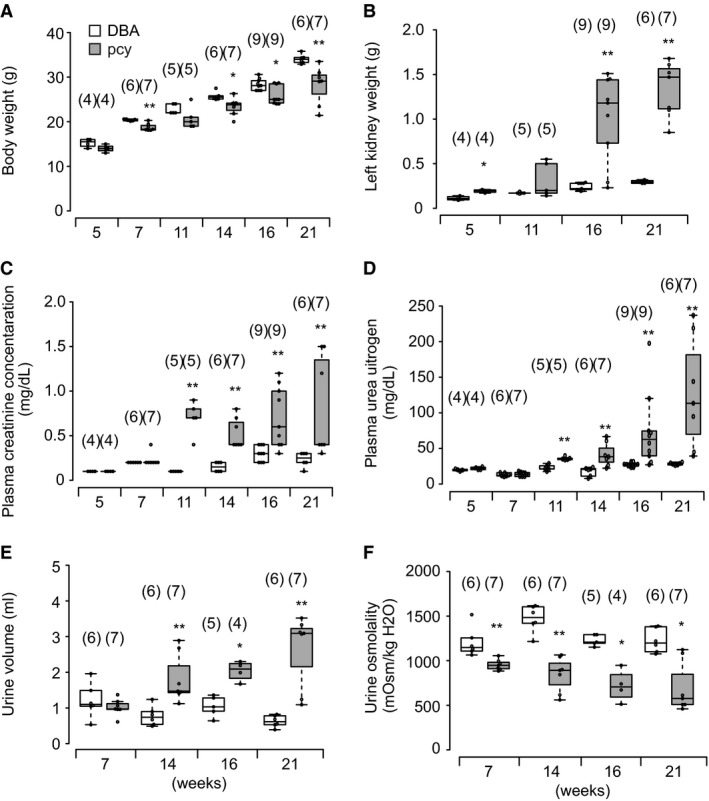
Basal characteristics of pcy mice. Body weight (A), left kidney weight (B), plasma creatinine (C) and urea nitrogen (D) concentrations, urine volume (E), and urinary osmolality (F) in the control DBA/2 (DBA) and DBA/2‐FG pcy (pcy) mice at the indicated weekly ages. Data are expressed as dot and box plots. Numbers in parentheses indicate the number of animals tested. *P < 0.05 and **P < 0.01, for comparison between DBA and pcy mice.

Figure 1A–F show the age‐related changes in body weight (Fig. 1A), left kidney weight (Fig. 1B), plasma creatinine (Fig. 1C) and urea nitrogen (Fig. 1D) concentrations, urine volume (Fig. 1E), and urinary osmolality (Fig. 1F) in pcy and DBA mice. pcy mice showed lower body weight at 7 weeks or older and an apparently heavier weight of the left kidney at 16 weeks and older, in comparison with DBA mice. The plasma creatinine and urea nitrogen concentrations were significantly higher in pcy mice than in DBA mice at 11 weeks and older. Urine volume was significantly increased at 14 weeks and older, and urinary osmolality became progressively lower with age in pcy mice in comparison with DBA mice.

### Age‐related changes in the release of uEV‐AQP2

**Figure 2:**
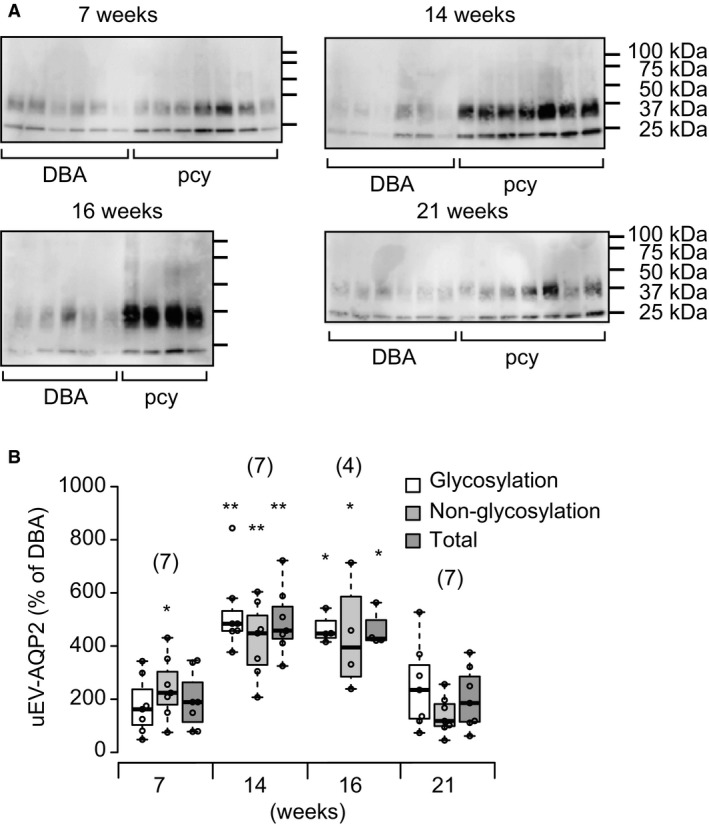
Release of uEV‐AQP2 in pcy mice. (A) Typical immunoblots of uEV‐AQP2 in DBA and pcy mice are shown. (B) Immunoblotting results were quantified and the summarized data are shown as dot and box plots. Each value is expressed as a percentage of the mean level in DBA mice at each time point. Glycosylation and nonglycosylation in B correspond to the upper and lower bands in each lane of A, respectively. Total indicates glycosylated + nonglycosylated AQP2. Numbers in parentheses indicate the numbers of animals tested. *P < 0.05 and **P < 0.01, for comparison between DBA and pcy mice.

The release of uEV‐AQP2 as judged by immunoblotting is shown in Figure 2. A slight increase in the release was observed in pcy mice at 7 weeks of age in comparison with DBA mice. Thereafter, the release increased markedly until 16 weeks. Interestingly, the level of the release in pcy mice subsequently decreased and did not differ significantly from that in DBA mice at 21 weeks. When we examined the glycosylated and nonglycosylated forms of AQP2 separately (Fig. 2B), the results obtained were essentially the same.

### Age‐related changes in the release of uEV‐TSG101 and ‐Alix

**Figure 3:**
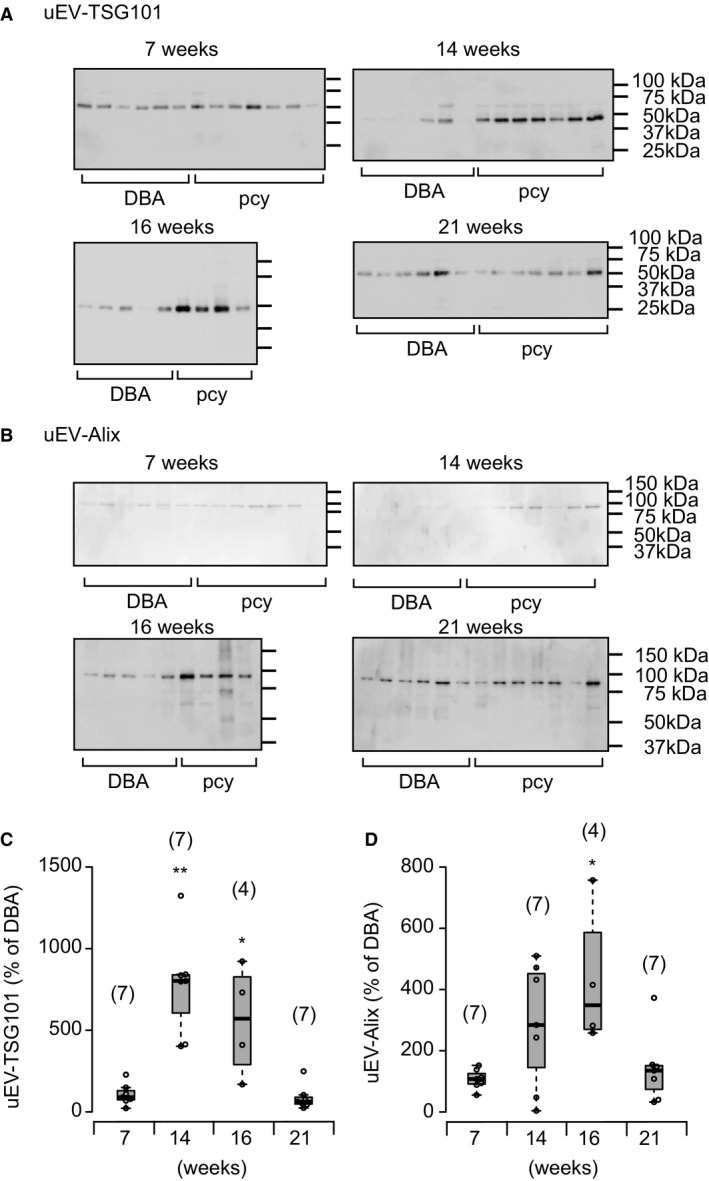
Release of uEV‐TSG101 and ‐Alix in pcy mice. (A) Typical immunoblots of uEV‐TSG101 in DBA and pcy mice. (B) Typical immunoblots of uEV‐Alix in DBA and pcy mice. Immunoblotting results were quantified and the summarized data for uEV‐TSG101 and uEV‐Alix are shown in (C) and (D), respectively. Data are expressed as dot and box plots. Each value is expressed as a percentage of the mean level in DBA mice at each time point. Numbers in parentheses indicate the numbers of animals tested. *P < 0.05 and **P < 0.01, for comparison between DBA and pcy mice.

Since TSG101 and Alix are known to be marker proteins for uEVs, especially exosomes (Erdbrügger and Le 2016; Oshikawa et al. 2016), we examined the release of uEV‐Alix and ‐TSG101 in pcy and DBA mice (Fig. 3). At both 7 and 21 weeks of age in pcy mice, the release of uEV‐TSG101 did not differ from that in DBA mice. On the other hand, in pcy mice at both 14 and 16 weeks, the release was significantly increased in comparison with DBA mice (Fig. 3A and C). Similarly, uEV‐Alix release was increased at 14 and 16 weeks in pcy mice, whereas the release at 7 and 21 weeks was not altered in comparison with DBA mice (Fig. 3B and D).

### Age‐related changes in renal expression of AQP2 protein

**Figure 4:**
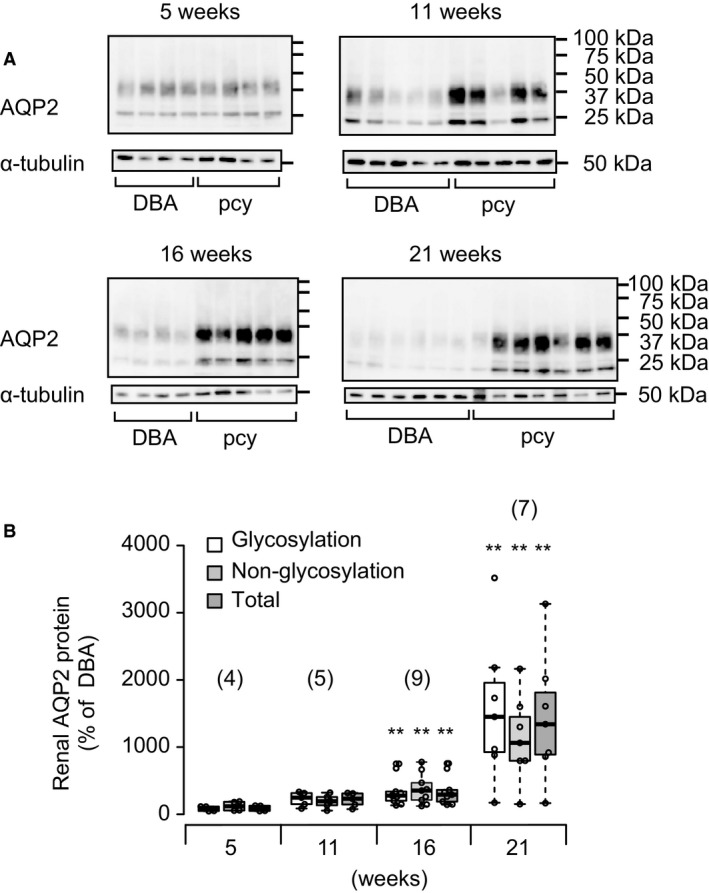
Renal expression of AQP2 in pcy mice. (A) Typical immunoblots of renal AQP2 and *α*‐tubulin. (B) Quantitative data were obtained from immunoblot analyses, and the summarized data for AQP2 after normalization to the corresponding level of *α*‐tubulin are shown as dot and box plots. Each value is expressed as a percentage of the mean level of renal expression of AQP2 in DBA mice at each time point. Data are expressed as dot and box plots. Numbers in parentheses indicate the numbers of animals tested. **P < 0.01, for comparison between DBA and pcy mice.

Next, we performed immunoblot analysis to examine the renal expression of AQP2. All quantitative data were normalized to *α*‐tubulin as an internal control. As shown in Figure 4, the expression level of renal AQP2 tended to be increased at 11 weeks of age and was significantly increased in pcy mice at 16 weeks and older in comparison with DBA mice. When we examined the glycosylated and nonglycosylated forms of AQP2 separately (Fig. 4B), the results obtained were essentially the same.

**Figure 5:**
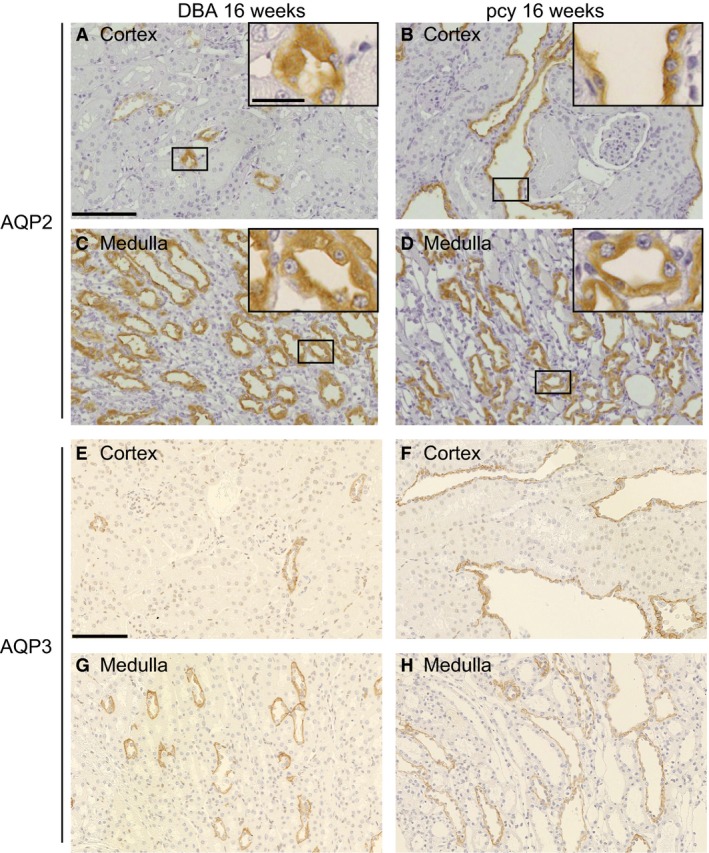
Immunohistochemistry of renal AQP2 in pcy mice at the age of 16 weeks. Kidney sections were stained with anti‐AQP2 (A–D) or anti‐AQP3 (E–H) antibody in DBA (A, C, E, and G) and pcy mice (B, D, F, and H). Representative images of the cortex (A, B, E, and F) and medulla (C, D, G, and H) are shown. The smaller black box in A–D indicates the region of the bigger black box in A–D. Brown staining indicates the presence of AQP2 or AQP3. Bars = 100 *μ*m. Bar (inset) = 20 *μ*m.

Figure 5A–D show the results of immunohistochemistry for AQP2 in pcy and DBA mice at 16 weeks of age. In the cortex, AQP2‐positive cells in cystic epithelial cells were markedly increased in pcy mice, although some cystic cells lacked AQP2 staining. The subcellular localization of AQP2 in pcy mice did not differ markedly from that in DBA mice (Fig. 5A–D, insets). On the other hand, in the medulla, the number of positive cells and the patterns of expression in pcy mice did not differ markedly from those in DBA mice.

AQP3 is known to be expressed in AQP2‐positive cells (Nielsen et al. 2002; Ikeda and Matsuzaki 2015). Therefore, we also performed immunohistochemistry with anti‐AQP3 antibody. As shown in Figure 5E–H, similarly to AQP2, AQP3‐positive cells were increased only in the cortex of pcy mice.

**Figure 6:**
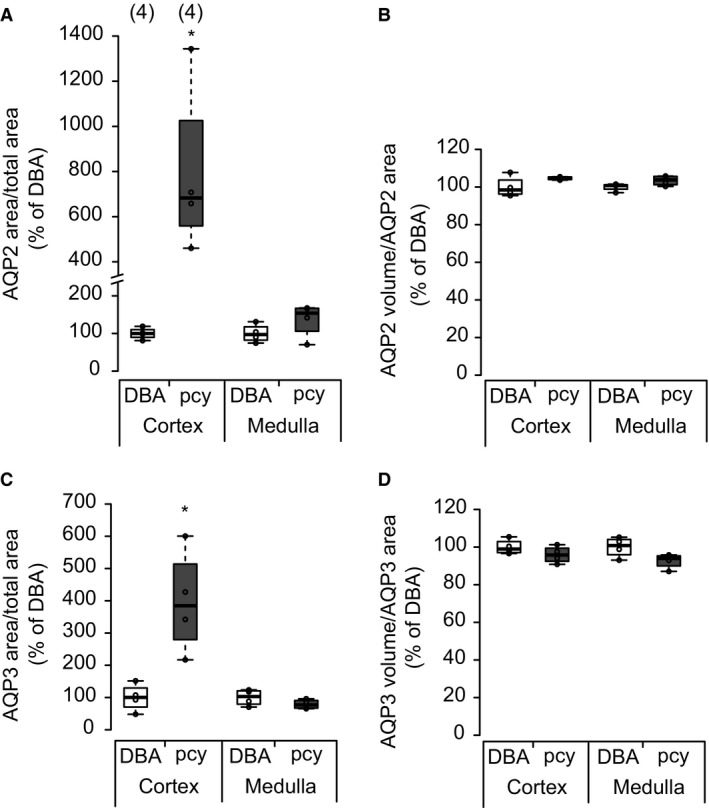
Semi‐quantitative immunohistochemistry data. Semi‐quantitative analysis was performed on immunohistochemistry images from 4 DBA and 4 pcy mouse kidneys. The areas and volumes of AQP2‐ and AQP3‐positive cells were measured (for details see Methods and Materials), and from these data the relative area and the density of AQP expression were calculated. The mean value for the DBA mouse group was taken as 100%. *P < 0.05 compared with the DBA mice.

In order to quantify the AQP2‐positive area and intensity of the expression, we analyzed the immunohistochemistry imaging data quantitatively. As shown in Figure 6, the AQP2‐positive area in the cortex, but not in the medulla was significantly larger in pcy mice than in DBA mice. On the other hand, the intensity of AQP2 expression in the cortex and medulla did not differ between pcy mice and DBA mice.

It was possibly thought that DBA mouse had a lower AQP2‐positive area than that in other normal mice species. Therefore, we compared AQP2‐positive area of DBA mice with that of C57BL/6J mice (4–5 weeks of age) in a separated experiment. As shown by a percentage of the mean level in C57BL/6J, the means ± SEM values of the AQP2‐positive area in the DBA mice cortex and medulla, were 122.7 ± 12.6% (*n* = 3) in the cortex and 79.3 ± 12.3% in the medulla, and there was no significant difference in each region between the two mice species.

We also quantified the AQP3‐positive area and intensity of the expression in pcy mice, and summarized the data in Figure 6. These data clearly showed similarities to the data for AQP2.

### Age‐related changes in renal expression of AQP mRNA

**Figure 7:**
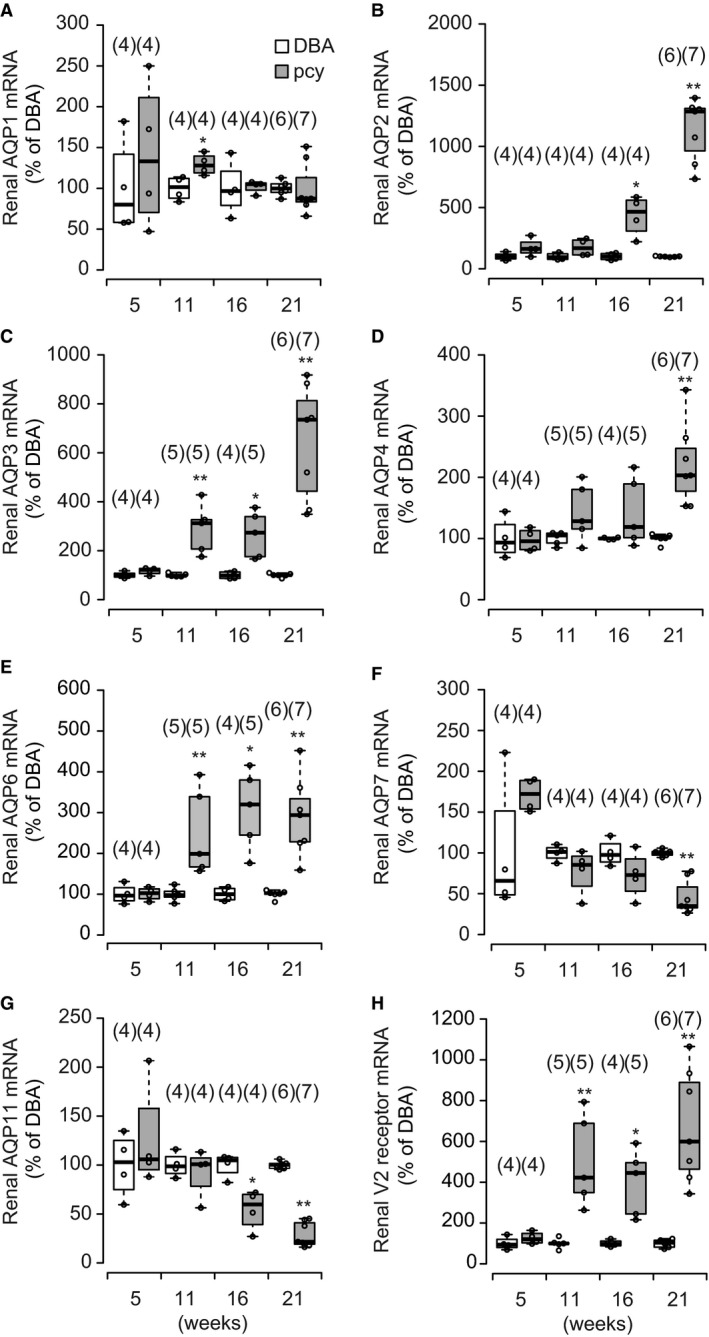
Age‐related changes in expression of renal AQPs and V2 receptor mRNAs in pcy mice. Renal AQP1, AQP2, AQP3, AQP4, AQP6, AQP7, AQP11 and V2 receptor mRNA levels in the whole kidney at the ages of 5, 11, 16, and 21 weeks in DBA and pcy mice were determined using a real‐time PCR assay. Each value is expressed as a percentage of the mean value of each mRNA in DBA mice. Data are expressed as dot and box plots. Numbers in parentheses indicate the numbers of animals tested. *P < 0.05 and **P < 0.01 compared with the control group.

If the increased abundance of renal AQP2 in pcy mice was attributable to an increased number of AQP2‐positive cells, then it would be expected that expression of AQP2 mRNA and mRNAs expressed in the same AQP2‐positive cells (principal cells of the collecting ducts), such as AQP3, AQP4, and V2 receptor mRNAs, would also be increased. Therefore, using a qPCR technique, we examined the expression of these AQPs and V2 receptor mRNAs as well as other AQP mRNAs expressed in cells other than principal cells (Nielsen et al. 2002; Ikeda and Matsuzaki 2015). As shown in Figure 7A, the level of AQP1 mRNA in pcy mice appeared to be constant at any age. The expression of AQP2, AQP3, AQP4, AQP6, and V2 receptor mRNAs was increased at 11 weeks or older in pcy mice. In contrast, the expression of AQP7 and AQP11 mRNAs decreased progressively after 16 weeks of age. These data suggested again that the area of the collecting ducts that expressed AQP2, AQP3, AQP4, AQP6, and V2 receptor mRNAs was enlarged in pcy mice.

### Relationship between uEV‐AQP2 and marker protein for uEV, or renal expression of AQP2

**Figure 8:**
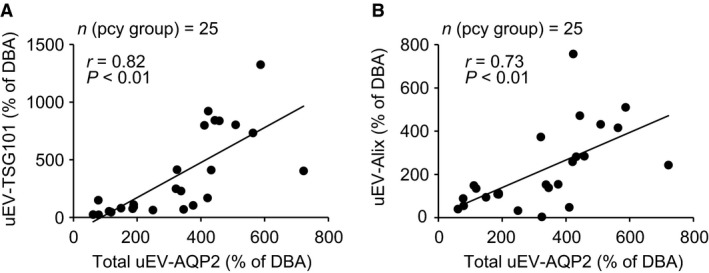
Relationship between uEV‐AQP2 and ‐TSG101, or ‐Alix. The line is the least‐squares regression line. The total (glycosylated + nonglycosylated forms) uEV‐AQP2 was used for this analysis.

Figures 2 and 3 suggested that the release of uEV‐AQP2 was related to the release of uEV marker proteins. Therefore, we evaluated the relationship between the release of the two. Figure 8 summarizes the data. There was a significant positive correlation between the release of either uEV‐AQP2 and ‐TSG101 or ‐Alix, suggesting that the level of uEV‐AQP2 depends on the number of EVs released into the urine.

**Figure 9:**
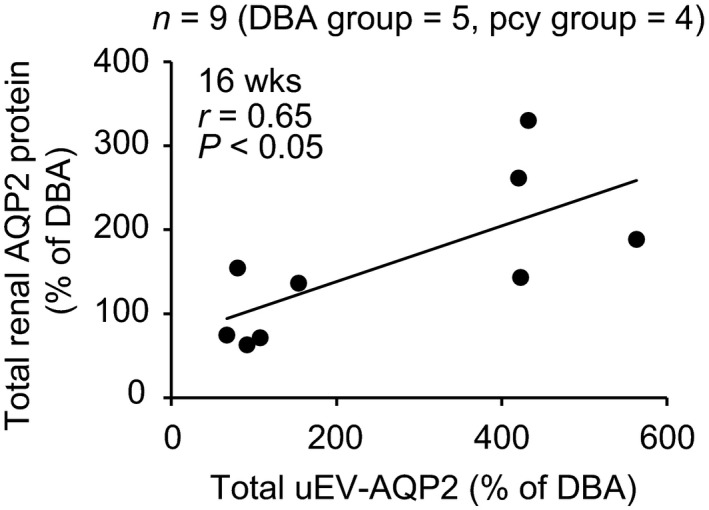
Relationship between AQP2 in the kidney and uEVs at the age of 16 weeks. The line is the least‐squares regression line. The total AQP2 (glycosylated + nonglycosylated forms) was used for this analysis.

Figures 2, 4, and 6 suggested that the release of uEV‐AQP2 was likely to be associated with the level of its renal expression at 16 weeks of age. Therefore, we examined the relationship between them. As shown in Figure 9, we found a significant positive correlation between the release of uEV‐AQP2 and the level of expression of renal AQP2.

## Discussion

In the present study, we have characterized for the first time the release of uEV‐AQP2 in pcy mice, an experimental model of NPHP. Renal failure with a defect in urinary concentration function was clearly observed in pcy mice after 11 weeks of age. The release of uEV‐AQP2 increased progressively until 16 weeks of age, but at 21 weeks the release in pcy mice was not significantly different from that in DBA mice (i.e. bell‐shaped). This release pattern resembled those of uEV‐TSG101 and ‐Alix. On the other hand, renal AQP2 expression increased progressively from 11 weeks, and there was a positive correlation between uEV‐AQP2 and uEV‐marker proteins. Also, at 16 weeks, there was a positive correlation between the release of uEV‐AQP2 and its level of renal expression. Because marker proteins have been shown to reflect the number of EVs released into urine (Lv et al. 2018), these data suggest that the level of uEV‐AQP2 depends on both the level of its renal expression and the number of EVs released into urine, at least in this experimental model of NPHP.

Our immunoblot analysis showed that both uEV‐TSG101 and ‐Alix were increased at around 14‐16 weeks of age, possibly reflecting the increased number of EVs released into urine. So far, although the mechanisms that regulate the release of EVs are still not entirely clear, some possibilities have been proposed. Park et al. (2010) have found that hypoxia facilitated the release of EVs from tumor cells. Furthermore, increased release of exosomes by breast cancer cells is reportedly mediated by the HIF1*α* pathway (King et al. 2012). On the other hand, the hypoxia‐HIF1*α* pathway is reportedly involved in cystic kidney injury (Bernhardt et al. 2007; Buchholz et al. 2014; Kraus et al. 2018). These findings suggest that the increases in the levels of uEV‐TSG101 and ‐Alix are mediated by the hypoxia‐HIF1*α* pathway. Alternatively, it has been reported that the increase in the release of EVs is related to the renal expression level of *α*‐smooth muscle actin (*α*‐SMA) in an initial phase of renal fibrosis (Asvapromtada et al. 2018). Since increased expression of *α*‐SMA has been reported in pcy mice (Okada et al. 2000), it is also possible that *α*‐SMA or initiation of renal fibrosis might be involved in the increased release of uEVs.

Immunoblot and qPCR analyses clearly showed that the levels of renal AQP2 protein and mRNA increased progressively in pcy mice. As mentioned earlier, this increase might contribute to the increase in the release of uEV‐AQP2 in pcy mice. It is considered that this increase was mediated by either an increase in the number of AQP2‐positive cells, an increase in the intensity of expression, or both. Immunohistochemistry revealed that AQP2‐positive cells were increased in the enlarged cystic region of the renal cortex in pcy mice at 16 weeks, whereas increased intensity of AQP2 expression was not obviously evident. qPCR analyses showed that the expression of AQP2 mRNA increased progressively from 11 weeks in pcy mice. This increase was accompanied by increases in AQP3, AQP4, AQP6, and V2 receptor mRNAs. For AQP3, the increase in AQP3‐positive cells in pcy mice was also observed by immunohistochemistry. AQPs and V2 receptors in the kidney are known to be expressed in a site‐specific manner, AQP1 being expressed in the proximal tubules, thin descending loop of Henle, and descending portion of the vasa recta, AQP2, AQP3, AQP4, and V2 receptor in the principal cells of collecting ducts, AQP6 in the *α*‐intercalated cells of collecting ducts, and AQP7 and AQP11 in the proximal tubules (Nielsen et al. 2002; Ikeda and Matsuzaki 2015). Therefore, the qPCR data strongly suggested that the region containing AQP2, AQP3, AQP4, AQP6, and V2 receptor mRNAs was enlarged in pcy mice. Together with these results, we thought that the increased expression of AQP2 in pcy mice was likely mediated by an increase in the number of AQP2‐positive cells, which in turn was related to enlargement of renal collecting ducts.

On the other hand, accordingly to the results of qPCR, the expression of AQP1 mRNA was not markedly altered and the expression of AQP7 and AQP11 mRNAs decreased progressively after 16 weeks in pcy mice. These three AQPs are expressed in the proximal tubules (Nielsen et al. 2002; Ikeda and Matsuzaki 2015). If the region of collecting ducts is selectively enlarged in pcy mice, the region of proximal tubules relative to the whole kidney would be decreased, and therefore expression of AQPs in the proximal tubules would be decreased. Although the decreased expression of AQP7 and AQP11 mRNAs supported this notion, the expression pattern of AQP1 mRNA did not. At present, there is no clear explanation for this. Since AQP1 is also known to be expressed in the thin descending loop of Henle and descending portion of the vasa recta (Nielsen et al. 2002; Ikeda and Matsuzaki 2015), expression in these regions might obscure the decrease in the expression of AQP1.

AQP6 is an AQP present in the intercalated cells where AQP2 is not expressed. Our qPCR results showed that expression of AQP6 mRNA was increased in pcy mice. This suggested that the population of AQP6‐positive intercalated cells was increased in the enlarged cystic region in pcy mice. In addition, immunohistochemistry demonstrated the presence of AQP2‐negative cells in the lining epithelium of the cysts. On the other hand, in experimental cystic kidney disease models, it has been reported that the cyst lining epithelium derived from collecting ducts contains only a minor population of intercalated cells (Shibazaki et al. 2008; Raphael et al. 2009). A future study, including accurate identification of the cell type present in the cyst epithelium of pcy mice, may clarify the reason for this difference.

We observed urinary concentration defects in pcy mice irrespective of increased expression of renal AQP2. It is known that water reabsorption by the vasopressin‐AQP2 system is driven by an osmotic gradient in the kidney and that a defect in the osmotic gradient disturbs water reabsorption (Verkman 1999; Sands et al. 2016). In fact, it has been pointed out that an insufficient osmotic gradient might be involved in the urinary concentration defects in cystic kidney disease (Krishnan et al. 2008). Therefore, we roughly measured tissue osmolality in the renal cortex and medulla in pcy mice using a reported method (Herrera and Garvin 2005). The tissue osmolality of the renal cortex in control DBA mice at 16 weeks of age was significantly lower than that of the renal medulla (cortex, 291.4 ± 11.6, *n* = 5; medulla, 381.8 ± 21.0 mOsm/kg, *n* = 5). In contrast, there was no significant difference in osmolality between the cortex and the medulla in age‐matched pcy mice (cortex, 320.8 ± 3.9, *n* = 5; medulla, 338.6 ± 12.6 mOsm/kg, *n* = 5). This suggested that the medullary osmotic gradient was disturbed in pcy mice, and that this might contribute to insufficient water reabsorption, irrespective of the increased expression of renal AQP2.

In summary, the age‐related release of uEV‐AQP2 in pcy mice showed a bell‐shaped pattern. This pattern was thought to be attributable to the numbers of renal AQP2‐positive cells and/or the numbers of EVs released into the urine.

## Conflict of Interest

None declared.
